# Does Regular Surveillance Improve the Long-Term Survival of Arteriovenous Fistulas?

**DOI:** 10.1155/2012/539608

**Published:** 2011-12-01

**Authors:** Ashwin Shetty, William L. Whittier

**Affiliations:** Division of Nephrology, Department of Internal Medicine, Rush University Medical Center, Chicago, IL 60607, USA

## Abstract

The rate of arteriovenous fistula (AVF) placement continues to rise and AVF failure is a major complication. The main cause of AVF failure is stenosis leading to thrombosis. Although the detection of early stenosis with preemptive correction prior to thrombosis seems to be a plausible option to prevent access failure, there is much debate, on the basis of studies of surveillance with arteriovenous grafts, as to whether early surveillance actually improves the longevity of AVFs. Evaluating the available information for surveillance, specifically the data for AVF stenosis and survival, is necessary to determine if surveillance is warranted. These trials have shown that vascular access flow (Qa) surveillance is beneficial in revealing subclinical stenosis. Preemptive angioplasty and surgical revision have shown to decrease thrombosis rates. However, at the present time, there is only limited data on whether preemptive treatment equates to improved long-term AVF survival.

## 1. Introduction

The occurrence of end-stage renal disease (ESRD) and dialysis access placement in the United States continues to increase [1]. Due to the superior long-term patency, lower complications, and decreased mortality rates, arteriovenous fistulas (AVFs) are the access of choice and represent 41.3% of the total hemodialysis accesses in the United States [1, 2]. Even as the rate of fistula placement improves, hemodialysis access failure continues to be a leading cause of hospitalizations and morbidity in the dialysis population [1]. The development of significant stenosis leading to poor flow and thrombosis is a leading cause of AVF revision and failure [2]. Although the detection of early stenosis with preemptive surgical correction or angioplasty prior to thrombosis seems to be a plausible option to prevent access failure, there is much debate as to whether early surveillance actually improves the longevity of AVFs.

There are many noninvasive methods available for the surveillance of stenosis (Table 1). Clinical monitoring is a useful technique that involves physical examination of the access site, excessive bleeding from the AVF venopuncture site, difficult cannulation, or unexplained reduction of urea reduction ratio (URR). Other surveillance methods are available including access recirculation, flow studies, pressure studies and direct visualization by Doppler ultrasonography [3]. These various methods of surveillance have been studied and are all predictive of stenosis [4, 5]. This has led to the guidelines by the National Kidney Foundation Dialysis Outcomes Quality Initiatives (K/DOQI) advocating routine surveillance of AVFs and arteriovenous grafts (AVGs) in dialysis centers [6].

Surveillance of AVGs and AVFs is defined separately by the K/DOQI guidelines and studies have shown different results in these two distinct access groups [6]. In AVGs, there has been considerable debate on the validity of surveillance for thrombosis. Initial observational studies revealed a reduction in graft thrombosis with routine surveillance programs and preemptive angioplasty [7]. However, this was not confirmed by randomized controlled trials, as surveillance and preemptive angioplasty failed to lower thrombosis rates or improve long-term graft survival [7]. Therefore, the recommendation of routine surveillance for AVGs [6] has been questioned [3].

Debate also exists surrounding surveillance for AVFs on eventual thrombosis or fistula longevity. Measurement of vascular access flow (Qa) is the recommended method of surveillance for AVFs, and much of this data is based on the observational studies in AVGs [6]. There have been numerous observational studies with historical control groups evaluating the benefit of Qa surveillance in AVFs, and results have been mixed [8–13]. Few of these studies [10–13] showed decreased thrombosis rates, while others [8, 9] showed no improvement [14]. Evaluating the available randomized controlled trials can better assess the question of whether the use of surveillance leads to a significant decrease in the rate of AVF thrombosis and improves long-term survival.

## 2. Randomized Controlled Trials

To date, there are four randomized trials that evaluated surveillance of AVF for stenosis and two of these evaluated the effect of prophylactic angioplasty on thrombosis rates and long-term outcomes (Table 2). In the most recent study, investigators randomly assigned 137 patients with AVFs to two groups: a group of monthly surveillance with Qa and clinical criteria and a control group with clinical criteria alone [15]. The clinical criteria consisted of an increase in dynamic venous pressures, decrease in blood flow (Qb), excessive bleeding from venopuncture sites or unexplained reduction in URR. The patients in the Qa surveillance arm were referred to angiography if their Qa was <500 mL/min or if it fell by >20% once the access flow was <1,000 mL/min. Each group was referred for any changes in clinical criteria. The primary end point was time to detection of a stenotic lesion that was ≥50%. Although thrombosis rates and AVF longevity were not reported in this study, the patients in the Qa surveillance arm were twice as likely to have stenosis detected compared to the control group.

The more relevant outcome of thrombosis rates were evaluated in a separate trial [16]. This study [16] included 103 patients of whom 68 had AVFs and 35 had AVGs. They were randomized into two major groups: one with monthly surveillance by Qa or static venous pressure and a control group. All patients underwent color flow Doppler ultrasound every 6 months. Patients with Qa < 750 mL/min or static venous pressure ratios ≥0.5 were referred for angiography. Using Doppler ultrasound, AVFs with >50% stenosis, Qa < 600 mL, or greater than 25% decline in Qa were also referred for angiography. If there was evidence of a stenotic lesion ≥50% of the vessel diameter, the patients underwent angioplasty. The primary end point was AVF thrombosis. Access longevity was not studied, but patients with AVFs followed with monthly surveillance by Qa and/or static venous pressure had a lower total thrombosis rate than the control patients (16.8 versus 27.1 per 100 patient years; *P* < 0.05).

 These studies revealed that surveillance using Qa detects early stenosis and leads to decreased thrombosis rates, but is there any long-term benefit for early intervention in patients with subclinical stenotic AVFs? This was evaluated in a trial [17] that included 60 patients with a stenotic lesion of >50% by angiogram who were randomized into a treatment group that underwent percutaneous transluminal angioplasty (PTA) or a control group with no intervention. Patients were initially screened with measurements of blood flow (Qb decrease by >30 mL/min on two consecutive hemodialysis sessions), access flow (Qa < 850 mL/min) or urea-based access recirculation. If there was an abnormality, these patients were sent for fistulography, and if >50% stenosis was found, they underwent randomization. All the AVFs with stenotic lesions were considered to be functional if they were providing adequate dialysis. The study end point was thrombosis or surgical revision due to inadequate dialysis. Impressively, the median functional failure-free AVF survival was 84 months (51.8 to 116.2 months) in the PTA group and 21 months (9.8 to 32.2 months) in controls (*P* < 0.001). A total of 6 patients in the PTA group had thrombosis compared to 14 patients in the control group (*P* = 0.029). However, the proportion of patients undergoing elective surgery was comparable. Therefore, early intervention in stenotic AVFs led to decreased time to and rates of thrombosis.

Surveillance of AVFs and early intervention has been shown to be successful in detecting stenosis and preventing thrombosis, but does identifying a significant stenosis lead to an increase in longevity of the access? A follow-up study from the same institution was performed shortly after in 2004 [18]. This study design was similar to the prior study. It included 79 patients with a significantly stenotic lesion who were randomized into treatment (PTA or surgical revision) or control group. The stenotic lesion was identified as in the above study with the only difference now being a Qa < 750 mL/min. One unique variation in the study protocol was that the treatment and control group were further divided into a “functional” (Qa > 350 mL/min) or “failing” (Qa ≤ 350 mL/min) group. The primary end point was primary patency as defined by the interval from stenosis to access failure. The primary patency rates were higher in the treatment groups compared to the control for both functional (*P* = 0.021) and failing subgroups (*P* = 0.005). Access survival rates were significantly higher in the treatment group than the control group (*P* = 0.050). Interestingly, within the treatment group, survival rates were also higher in the functional subgroup compared to the failing one (*P* = 0.033). This study showed that early intervention on stenotic AVFs leads to increased longevity. If intervention is delayed until Qa flow is too low, then the AVF was not as salvageable with intervention.

 When, then, is the appropriate time to intervene? Should prophylactic correction of subclinical stenosis become universal when a low Qa is detected? The two trials that evaluated preemptive treatment of subclinical stenosis showed decreased thrombosis rates [17, 18]. In both trials, the majority of patients were sent to angiography, because their Qa was <850 mL/min or 750 mL/min, further validating the importance of using a surveillance program. The current guidelines recommend correction only in poorly functioning AVFs that are causing clinical compromise [6]. These trials show that preemptive treatment of stenosis detected by surveillance, prior to clinical consequences, is beneficial and in one trial [18], preemptive treatment was better when the Qa > 350 mL/min. Once the Qa became lower than this threshold, the access was past the point of repair.

One of the major limitations of universally recommending preemptive intervention is that there is a paucity of information on long-term survival, as only one study evaluated this [18]. Even in that study, over a quarter of the treatment group underwent surgical revision as opposed to PTA [19] that may have contributed to these AVFs surviving longer. In fact, one study [16] revealed an increased restenosis rate after PTA.

The key question of whether detection of stenosis and early intervention increases the longevity of AVFs is unfortunately still not answered. Even though thrombosis rates have been lower in all studies, if there is no correlation to longer AVF survival, the beneficial effect may be inconsequential. If there is no benefit of access survival with surveillance, increased studies and interventions only consume more resources and add to cost. The method of repair (PTA or surgical) is also relevant. Although the available information suggests a benefit with surveillance, there needs to be larger, powered randomized trials on the long-term survival of AVF to conclusively settle the issue of the use of surveillance leading to longer AVF survival.

## 3. Conclusion

Observational studies have shown mixed results for the use of Qa surveillance in preventing AVF thrombosis. Randomized controlled trials, though, have shown that Qa surveillance is beneficial in revealing subclinical stenosis. Preemptive angioplasty and surgical revision have shown to decrease thrombosis rates. Larger, randomized controlled trials need to be done to show whether preemptive treatment equates to improved long-term AVF survival.

## Figures and Tables

**Table 1:** Noninvasive methods of surveillance for AV access stenosis.

Clinical monitoring
Access recirculation
Flow studies
Sodium, urea, glucose, differential conductivity, inline dialysance, ultrasound dilution and thermal methods
Pressure studies
Dynamic
Static
Direct visualization
Doppler ultrasonography
Magnetic resonance angiography

**Table 2:** Randomized controlled trials of arteriovenous fistula surveillance.

Name	Survey method	Preemptive angioplasty/surgical revison	Control group	Treatment group	Reduce thrombosis	Prolong survival
Polkinghorne et al.[15]	Qa (<550 mL/min) and Clinical criteria versus Clinical criteria alone	No	68	69	*	*
Sands et l. [16]	Qa (<800 mL/min), Static venous pressure and Doppler ultrasound versus Doppler ultrasound alone	No	40	63	Yes	*
Tessitore et al. [17]	Qa (<850 mL/min), Qb, Ru, and Rhd	Yes	30	32	Yes	*
Tessitore et al. [18]	Qa (<750 mL/min), Qb, and Ru	Yes	36	43	Yes	Yes

*Data/values not reported or unavailable.

Qa: vascular access flow, Qb: blood flow, Ru: urea-based access recirculation, and Rhd: ultrasound dilution recirculation.
